# Characterization of a bacteriophage, vB_Eco4M-7, that effectively infects many *Escherichia coli* O157 strains

**DOI:** 10.1038/s41598-020-60568-4

**Published:** 2020-02-28

**Authors:** Agnieszka Necel, Sylwia Bloch, Bożena Nejman-Faleńczyk, Michał Grabski, Gracja Topka, Aleksandra Dydecka, Katarzyna Kosznik-Kwaśnicka, Łukasz Grabowski, Agata Jurczak-Kurek, Tomasz Wołkowicz, Grzegorz Węgrzyn, Alicja Węgrzyn

**Affiliations:** 1 Department of Molecular Biology, Faculty of Biology, University of Gdańsk, Wita Stwosza 59, 80-308 Gdańsk, Poland; 2 Laboratory of Molecular Biology, Institute of Biochemistry and Biophysics, Polish Academy of Sciences, Kładki 24, 80-822 Gdańsk, Poland; 3 Department of Molecular Evolution, Faculty of Biology, University of Gdańsk, Wita Stwosza 59, 80-308 Gdańsk, Poland; 4 Department of Bacteriology and Biocontamination Control, National Institute of Public Health-National Institute of Hygiene, Chocimska 24, 00-791 Warsaw, Poland

**Keywords:** Bacteriophages, Phage biology

## Abstract

The characterization of a recently isolated bacteriophage, vB_Eco4M-7, which effectively infects many, though not all, *Escherichia coli* O157 strains, is presented. The genome of this phage comprises double-stranded DNA, 68,084 bp in length, with a GC content of 46.2%. It contains 96 putative open reading frames (ORFs). Among them, the putative functions of only 35 ORFs were predicted (36.5%), whereas 61 ORFs (63.5%) were classified as hypothetical proteins. The genome of phage vB_Eco4M-7 does not contain genes coding for integrase, recombinase, repressors or excisionase, which are the main markers of temperate viruses. Therefore, we conclude that phage vB_Eco4M-7 should be considered a lytic virus. This was confirmed by monitoring phage lytic development by a one-step growth experiment. Moreover, the phage forms relatively small uniform plaques (1 mm diameter) with no properties of lysogenization. Electron microscopic analyses indicated that vB_Eco4M-7 belongs to the *Myoviridae* family. Based on mass spectrometric analyses, including the fragmentation pattern of unique peptides, 33 phage vB_Eco4M-7 proteins were assigned to annotated open reading frames. Importantly, genome analysis suggested that this *E. coli* phage is free of toxins and other virulence factors. In addition, a similar, previously reported but uncharacterized bacteriophage, ECML-117, was also investigated, and this phage exhibited properties similar to vB_Eco4M-7. Our results indicate that both studied phages are potential candidates for phage therapy and/or food protection against Shiga toxin-producing *E. coli*, as the majority of these strains belong to the O157 serotype.

## Introduction

The appearance of multidrug-resistant bacterial strains, particularly human pathogens, is one of the major problems of current medicine^1^. Bacteriophage therapy (or phage therapy) is one possible alternative to treat bacterial infections^2–5^. This potential therapeutic option is based on the assumption that bacteriophages, i.e., viruses that can destroy bacterial cells, can infect and eliminate bacterial pathogens in humans or animals^5^. Bacteriophages are usually specific to a single bacterial species, or even strain, and they are able to propagate only if specific host bacteria are available. Moreover, since phages do not infect eukaryotic cells, they are considered safe for use in the treatment of humans and animals^4^.

Although phage therapy is a promising alternative to the use of antibiotics, there are several factors that limit the utility of this therapeutic method^6^. First, a large collection of different phages is required to provide adequate therapeutic options for many patients suffering from different infectious diseases. Second, not every phage is suitable for phage therapy. In particular, temperate phages should not be used in this procedure because they can lysogenize host cells instead of lyse them. Third, some phages carry toxin genes in their genomes and therefore cannot be used as therapeutic agents. Fourth, it is possible for bacteria to develop resistance to phages.

Despite the limitations described above, the antibacterial activities of bacteriophages are very attractive in combating the presence of unwanted bacteria. Hence, apart from employing them in the treatment of human diseases, the use of these viruses is also considered in food protection^7^, agriculture and industry^3^.

Although the vast majority of *Escherichia coli* strains are human commensals, there are also pathogenic strains belonging to this species. Among them, Shiga toxin-producing *E. coli* (STEC), including the enterohemorrhagic *E. coli* (EHEC) group, appear to be some of the most dangerous pathogens^8^. While the first symptoms of infections by these bacteria appear to be unpleasant but not dangerous (bloody diarrhoea), complications, such as haemolytic uremic syndrome, thrombocytopenia and haemorrhagic colitis, can cause severe symptoms or even death in patients^9,10^. In fact, local epidemies caused by STEC can be tragic, such as that in 2011 in Germany, which caused over 50 deaths^11,12^. The treatment of STEC infection is particularly difficult, as Shiga toxin production is induced in the presence of various antibiotics; thus, some antibiotics cannot be used to treat STEC infections^13^. Therefore, phage therapy presents a promising alternative to the antibiotic treatment of STEC infections. The majority of STEC strains belong to the O157:H7 serotype^8,9,13^; thus, isolation and characterization of phages infecting such bacteria is potentially useful for further studies on their use in phage therapy and/or food protection. In this report, we describe the characterization of the newly isolated bacteriophage vB_Eco4M-7, which effectively infects many, though not all, *E. coli* O157 hosts.

## Results and Discussion

### The vB_Eco4M-7 bacteriophage

The vB_Eco4M-7 phage was isolated from urban sewage, and it was included in the list of newly isolated coliphages described recently^14^. Here, we present a detailed characterization of this bacteriophage.

### Basic characteristics of the vB_Eco4M-7 genome

**Figure 1:**
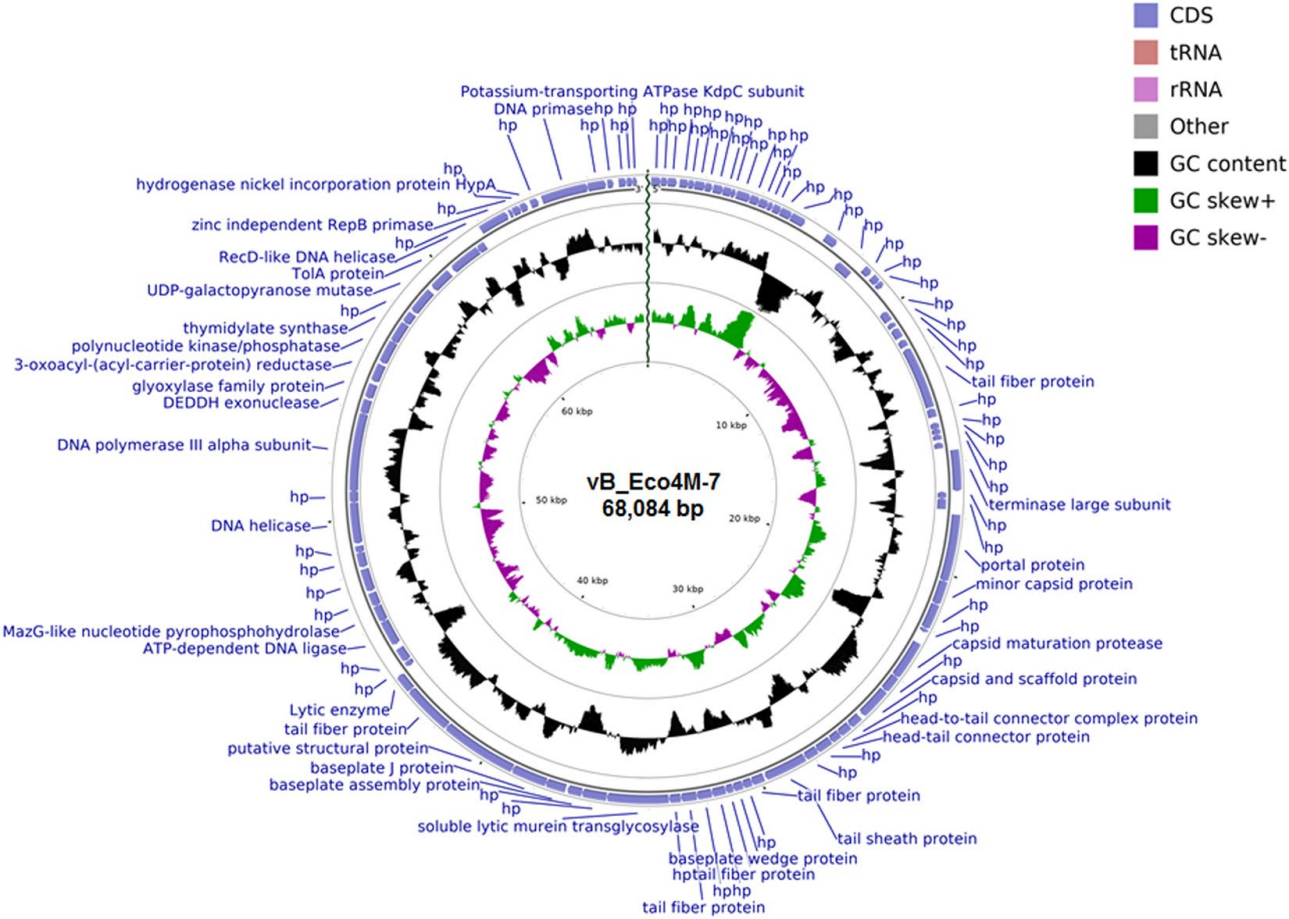
Map of the genome organization of bacteriophage vB_Eco4M-7 created by using the BRIG platform and the CGView program. The ORFs with predicted annotations are indicated with blue arrows. The inner ring shows genome location, GC skew + (green), GC skew − (purple) and GC content (black).

The full nucleotide sequence of the genome of *E. coli* virus vB_Eco4M-7 has been determined, and this sequence has been deposited (the NCBI accession number is MN176217). The vB_Eco4M-7 genome is composed of double-stranded DNA, 68,084 bp in length, with a GC content of 46.2% (Fig. 1). The annotation information, such as positions, directions, and functions, of each gene and conserved protein domains of phage vB_Eco4M-7 are summarized in Supplementary Table S1.

Bioinformatic analyses of the vB_Eco4M-7 genome indicated that it contains 96 putative open reading frames (ORFs), from which 62 ORFs were located on the direct strand of the virus genome and 34 ORFs were on the complementary strand (Fig. 1 and Supplementary Table S1). Among all identified ORFs, 35 were assigned putative functions (36.5%) based on their amino acid sequence homology to known proteins or evolutionarily conserved protein domains and motifs. Approximately 63.5% of ORFs (61 ORFs) were classified as hypothetical proteins of unknown function (Fig. 1 and Supplementary Table S1). The genome of phage vB_Eco4M-7 does not contain sequences of genes encoding integrase, recombinase, repressors and excisionase, which are the main markers of temperate viruses. Consequently, the vB_Eco4M-7 phage should be considered a lytic virus. Furthermore, the results obtained after genetic screening of the vB_Eco4M-7 genome against the Virulence Factors of Pathogenic Bacteria database suggest that this *E. coli* phage is free of genes coding for toxins and other virulence factors that might affect eukaryotic cells. This is an important indication as detecting any of the virulence-associated genes by genetic screening would immediately disqualify vB_Eco4M-7 phage for therapeutic use.

**Figure 2:**
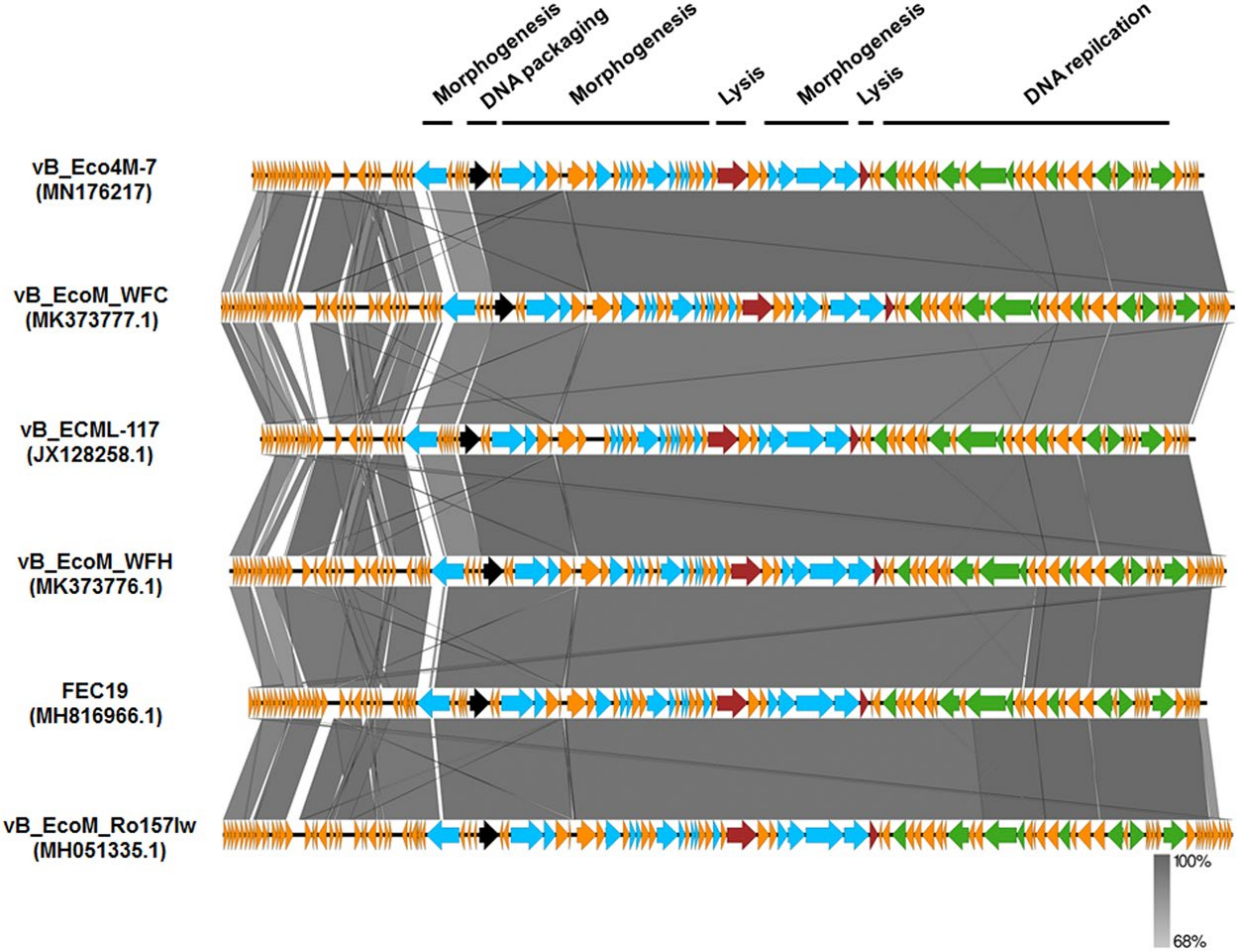
Schematic genomic alignment of phage vB_Eco4M-7 with 5 other related phages generated by using the EasyFig program. GenBank accession numbers of genomes of the bacterial viruses are given in brackets. Arrows with different colours represent ORFs associated with the genomic regions indicated at the top. The grey bar in the lower right corner shows the identity percentage associated with the colour of the bars connecting homologous regions.

**Figure 3:**
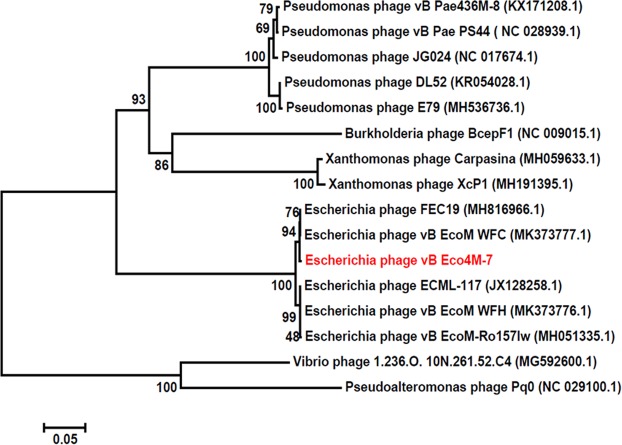
Phylogenetic tree based on the amino acid sequence of the terminase large subunit (TerL) showing the position of bacteriophage vB_Eco4M-7 (coloured red). The alignment of sequences was performed by using MUSCLE. The tree was generated by using MEGA7 and the neighbour-joining method with 1,000 bootstrap replicates. The bootstrap values are shown at the nodes.

As shown in Figs. 2 and 3, the large subunit of the terminase of phage vB_Eco4M-7 is closely related to large terminase subunits of other virulent *Myoviridae* bacteriophages that infect *E. coli*. Based on the results of BLAST analyses, the sequence of the genome of the vB_Eco4M-7 virus displays significant similarity (coverage 94–96%, identity 92–97%) to 5 phages: vB_EcoM_WFC (MK373777.1), vB_ECML-117 (JX128258.1), vB_EcoM_WFH (MK373776.1), FEC19 (MH816966.1) and vB_EcoM_Ro157Iw (MH051335.1). In addition, the gene inventories of these 6 closely related phages are highly similar. As indicated in Fig. 2, genomes of all tested phages contain blocks of genes categorized with similar functions and mechanisms of action, such as proteins responsible for DNA replication (ATP-dependent DNA ligase, DNA helicase, alpha subunit of DNA polymerase III, DEDDH exonuclease, thymidylate synthase, RecD-like DNA helicase, DNA primase), morphogenesis (tail fibre proteins, minor head and capsid proteins, scaffold proteins, head-tail connector proteins, tail sheath proteins, baseplate wedge proteins), DNA packaging (large subunit of terminase) and host lysis (soluble lytic murein transglycosylase, lytic enzyme). Interestingly, multiple alignment of phage vB_Eco4M-7 and 5 phage relatives showed differences in the sequences of the vB_Eco4M-7_27 gene encoding the tail fibre protein, which is thought to be involved in host recognition. This incompatibility may make the host range of phage vB_Eco4M-7 different from other closely related bacteriophages.

### Identification of phage proteins by using mass spectrometry

The mass-spectrometric (MS) identification of phages vB_Eco4M-7 and ECML-117 (a previously described phage similar to vB_Eco4M-7) proteins was based on the fragmentation pattern of unique peptides. The obtained results are presented in Tables 1 and 2. The MS data provided information about the molecular mass of the detected proteins, the number of unique peptides, the sequence coverage and the protein score. On this basis, we assigned 33 vB_Eco4M-7 phage proteins and 28 ECML-117 phage proteins to annotated open reading frames (ORFs). In this manner, protein annotations of 13 *in silico*-predicted structural proteins of vB_Eco4M-7 (vB_Eco4M-7_27, vB_Eco4M-7_36, vB_Eco4M-7_37, vB_Eco4M-7_42, vB_Eco4M-7_45, vB_Eco4M-7_48, vB_Eco4M-7_49, vB_Eco4M-7_51, vB_Eco4M-7_52, vB_Eco4M-7_60, vB_Eco4M-7_61, vB_Eco4M-7_62, vB_Eco4M-7_63) were confirmed. Interestingly, the capsid maturation protease (vB_Eco4M-7_40), DNA helicase (vB_Eco4M-7_73) and glyoxalase family protein (vB_Eco4M-7_77) were also determined in phage vB_Eco4M-7. In the case of phage ECML-117, only 3 *in silico*-predicted structural proteins were detected by using MS (PI34_gp24, PI34_gp35 and PI34_gp40). The presence of putative 3-oxoacyl reductase (PI34_gp76) in ECML-117 lysate was also confirmed. Moreover, the results of MS analysis indicated that both tested phages produce transglycosylase (vB_Eco4M-7_57, PI34_gp55), lytic enzyme (vB_Eco4M-7_64, PI34_gp62), and TolA protein (vB_Eco4M-7_83, PI34_gp81). Additionally, 14 gene products of vB_Eco4M-7 and 21 gene products of ECML-117 having no similarities to known phage proteins could be classified as proteins of unknown function. The identification analyses of the proteins are verified in Supplementary Tables S2 and S3.

**Table 1:** Mass spectrometry data for bacteriophage Eco4M-7.

Detected proteins	Predicted function	Molecular mass (kDa)	Number of peptides	Sequence coverage (%)	Protein Score
vB_Eco4M-7_09	Unknown protein	11.7	2	26.42	16.68
vB_Eco4M-7_16	Unknown protein	15.17	2	24.46	4.64
vB_Eco4M-7_19	Unknown protein	23.0	4	31.73	66.43
vB_Eco4M-7_26	Unknown protein	10.5	5	62.26	254.06
vB_Eco4M-7_27	Tail fibre protein	82.7	34	80.23	7387.37
vB_Eco4M-7_32	Unknown protein	7.8	3	55.26	1002.01
vB_Eco4M-7_36	Portal Protein	88.7	20	34.26	1112.31
vB_Eco4M-7_37	Minor capsid protein	31.7	11	44.17	192.14
vB_Eco4M-7_38	Unknown protein	30.9	8	72.88	3353.70
vB_Eco4M-7_40	Capsid maturation protease	51.1	28	58.30	5402.22
vB_Eco4M-7_41	Unknown protein	21.4	6	37.56	2231.54
vB_Eco4M-7_42	Capsid and scaffold protein	41.7	15	54.88	936.28
vB_Eco4M-7_45	Head-tail connector protein	14.9	2	18.75	16.32
vB_Eco4M-7_46	Unknown protein	21.9	11	70.16	331.31
vB_Eco4M-7_47	Unknown protein	18.1	3	29.81	114.45
vB_Eco4M-7_48	Tail sheath protein	54.8	12	33.59	891.46
vB_Eco4M-7_49	Tail fibre protein	16.5	6	52.26	282.76
vB_Eco4M-7_51	Baseplate wedge protein	12.8	6	45.76	163.14
vB_Eco4M-7_52	Tail fibre protein	9.8	10	64.77	2928.80
vB_Eco4M-7_53	Unknown protein	17.6	3	24.85	4.52
vB_Eco4M-7_54	Unknown protein	19.6	3	15.82	9.47
vB_Eco4M-7_57	Soluble lytic murein transglycosylase	79.0	30	53.86	392.27
vB_Eco4M-7_58	Unknown protein	32.4	8	39.31	138.95
vB_Eco4M-7_60	Baseplate assembly protein	25.4	12	79.25	774.20
vB_Eco4M-7_61	Baseplate J protein	45.1	9	43.26	211.87
vB_Eco4M-7_62	Putative structural protein	98.6	13	22.15	182.68
vB_Eco4M-7_63	Tail fibre	63.4	13	34.62	890.91
vB_Eco4M-7_64	Lytic Enzyme	22.9	2	12.15	5.69
vB_Eco4M-7_70	Unknown protein	31.2	12	40.21	518.15
vB_Eco4M-7_71	Unknown protein	20.7	6	48.11	623.32
vB_Eco4M-7_73	DNA helicase	58.6	2	5.52	2.17
vB_Eco4M-7_77	Glyoxalase family protein	20.8	8	49.73	718.37
vB_Eco4M-7_83	TolA protein	31.1	7	25.81	1197.60

**Table 2:** Mass spectrometry data for bacteriophage ECML-117.

Detected proteins	Predicted function	Molecular mass (kDa)	Number of peptides	Sequence coverage (%)	Protein Score
PI34_gp23	Unknown protein	7.6	2	28.99	2.25
PI34_gp24	Phage tail fibre protein	82.9	18	36.94	302.42
PI34_gp30	Unknown protein	7.7	2	27.63	95.98
PI34_gp34	Unknown protein	88.6	22	36.54	318.48
PI34_gp35	Phage minor capsid protein	31.8	10	35.69	31.57
PI34_gp36	Unknown protein	30,8	3	15.69	59.73
PI34_gp38	Unknown protein	51.1	21	39.79	2644.82
PI34_gp39	Unknown protein	21.5	5	35.12	194.80
PI34_gp40	Phage capsid and scaffold protein	41.7	12	29.82	109.86
PI34_gp41	Unknown protein	16,8	4	32.47	13.98
PI34_gp43	Unknown protein	14.9	2	15.63	4.62
PI34_gp44	Unknown protein	21.8	6	34.03	30.91
PI34_gp46	Unknown protein	46.0	4	11.27	20.84
PI34_gp47	Unknown protein	16.5	4	21.94	13.71
PI34_gp48	Unknown protein	12.4	2	45.87	4.06
PI34_gp49	Unknown protein	12.8	6	45.76	14.39
PI34_gp50	Unknown protein	9.8	3	28.41	12.32
PI34_gp55	Soluble lytic murein transglycosylase	78.9	24	40.08	64.82
PI34_gp56	Unknown protein	32.3	3	12.76	3.92
PI34_gp58	Unknown protein	25.5	5	26.56	17.38
PI34_gp59	Unknown protein	45.0	7	25.30	25.66
PI34_gp60	Unknown protein	98.6	17	26.43	64.84
PI34_gp61	Unknown protein	63.2	9	20.54	40.80
PI34_gp62	Lytic enzyme	23.0	12	69.16	93.79
PI34_gp68	Unknown protein	31,2	5	23,08	62,77
PI34_gp75	Unknown protein	20.7	5	33.88	19.47
PI34_gp76	Putative 3-oxoacyl reductase	24.9	3	45.87	4.06
PI34_gp81	TolA protein	31.1	2	8.24	3.21

### Host range of vB_Eco4M-7 and ECML-117

We tested the host range of vB_Eco4M-7 and the closely related phage ECML-117. Both phages could form plaques on lawns of 16 Shiga toxin-producing *E. coli* O157:H7 strains isolated from stool (Table 3). Moreover, we found that the tested phages were able to efficiently infect the following O157 strains: 3 *E. coli* O157:H7 non-STEC isolates from stool, 11 *E. coli* O157 STEC isolates from stool and food and 4 *E. coli* O157 non-STEC strains. Interestingly, phage vB_Eco4M-7 apparently did not infect *E. coli* O26 STEC isolates from stool or 41 other tested *E. coli* non-O157 strains, including non-pathogenic *E. coli* bacteria. However, phage ECML-117, in contrast to phage vB_Eco4M-7, formed plaques on 6 *E. coli* O25 strains. Other tested *E. coli* strains, both laboratory and enteropathogenic *E. coli* (EPEC) isolates, were resistant to the tested phages. Moreover, vB_Eco4M-7 and ECML-117 could not infect the tested *Shigella flexneri, Salmonella enterica, Bacillus* sp., *Pseudomonas aeruginosa, Enterococcus faecium, Staphylococcus aureus, Klebsiella* sp., and *Acinetobacter* sp. strains (Table 3). Therefore, we conclude that these phages are effective in infecting most *E. coli* O157 strains, including various *E. coli* O157:H7 strains, though some of these hosts remained resistant to these phages. This property is advantageous when considering vB_Eco4M-7 and ECML-117 as potential agents for phage therapy and/or food protection, as the specificity of phages can allow only one pathogenic *E. coli* strain to be targeted while leaving nonpathogenic bacteria undamaged.

**Table 3:** Host specificity of bacteriophages ECML-117 and vB_Eco4M-7.

Bacterial strain	ECML-117	vB_Eco4M-7
*Escherichia coli* MG1655	−	−
*Escherichia coli* C600	−	−
*Escherichia coli* Tap90	−	−
*Escherichia coli* Hfr3000	−	−
*Escherichia coli* MC1061	−	−
*Escherichia coli* DH5α	−	−
*Escherichia coli* EPEC-A	−	−
*Escherichia coli* EPEC-B	−	−
*Escherichia coli* EPEC-C	−	−
*Escherichia coli* CB571	−	−
*Escherichia coli* EDL933	−	−
*Escherichia coli* 3250	−	−
*Escherichia coli* 23580	−	−
*Escherichia coli* 23581	−	−
*Escherichia coli* O157:H7(ST2–8624)	+	+
*Escherichia coli* O157:H7 700728	+	+
*Escherichia coli* O157:H7 17076	+	++
*Escherichia coli* O157:H7 19206	+	+
*Escherichia coli* O157:H7 174/03	+	+
*Escherichia coli* O157:H7 598/03	+	+
*Escherichia coli* O157:H7 49/04	+	+
*Escherichia coli* O157:H7 365/05	+	+
*Escherichia coli* O157:H7 175/06	+	+
*Escherichia coli* O157:H7 206/06	+	+
*Escherichia coli* O157:H7 474/07	−	−
*Escherichia coli* O157:H7 371/08	+	+
*Escherichia coli* O157:H7 4/10	+	+
*Escherichia coli* O157:H7 251/10	+	+
*Escherichia coli* O157:H7 79/13	+	+
*Escherichia coli* O157:H7 242/13	+	+
*Escherichia coli* O157:H7 262/13	+	+
*Escherichia coli* O157:H7 224/14	+	+
*Escherichia coli* O157:H7 226/14	++	+
*Escherichia coli* O157:H7 58/17	+	+
*Escherichia coli* O157:H7 8185	−	−
*Escherichia coli* O157 225/96	+	+
*Escherichia coli* O157 440/98	+	+
*Escherichia coli* O157 19/99	+	+
*Escherichia coli* O157 568/99	+	+
*Escherichia coli* O157 286/00	+	++
*Escherichia coli* O157 214/04	+	+
*Escherichia coli* O157 443/07	+	+
*Escherichia coli* O157 131/17	+	+
*Escherichia coli* O157 99/18	−	−
*Escherichia coli* O157 4/19	+	+
*Escherichia coli* O157 345/96	+	+
*Escherichia coli* O157 346/96	−	−
*Escherichia coli* O157 347/96	+	+
*Escherichia coli* O157 42/16	+	+
*Escherichia coli* O157 95/16	+	+
*Escherichia coli* O157 99/16	++	++
*Escherichia coli* O157 156/16	+	+
*Escherichia coli* O157 18/19	−	−
*Escherichia coli* O157 13/17	−	−
*Escherichia coli* O25 191/19	+	−
*Escherichia coli* O25 49/19	+	−
*Escherichia coli* O25 13/19	−	−
*Escherichia coli* O25 171/18	+	−
*Escherichia coli* O25 170/18	−	−
*Escherichia coli* O25 169/18	−	−
*Escherichia coli* O25 84/17	−	−
*Escherichia coli* O25 45/16	+	−
*Escherichia coli* O25 43/16	+	−
*Escherichia coli* O25 191/15	−	−
*Escherichia coli* O26 113/19	−	−
*Escherichia coli* O26 214/15	−	−
*Escherichia coli* O44 254/15	−	−
*Escherichia coli* O44 166/15	−	−
*Escherichia coli* O55 22/17	−	−
*Escherichia coli* O86 149/17	−	−
*Escherichia coli* O111 89/15	−	−
*Escherichia coli* O119 137/18	−	−
*Escherichia coli* O125 162/19	−	−
*Escherichia coli* O126 10/18	−	−
*Escherichia coli* O127 60/17	−	−
*Escherichia coli* O127 53/17	−	−
*Escherichia coli* O128 145/17	−	−
*Escherichia coli* O128 55/17	−	−
*Escherichia coli* 185/19	−	−
*Escherichia coli* 135/19	−	−
*Escherichia coli* 111/19	−	−
*Escherichia coli* 16/19	−	−
*Escherichia coli* 3/19	−	−
*Escherichia coli* 93/16	−	−
*Escherichia coli* 91/16	−	−
*Escherichia coli* 90/16	−	−
*Escherichia coli* 68/16	−	−
*Escherichia coli* 65/16	−	−
*Escherichia coli* 12/16	−	−
*Escherichia coli* 296/15	−	−
*Escherichia coli* 246/15	−	−
*Escherichia coli* 146/15	−	−
*Escherichia coli* 144/15	−	−
*Escherichia coli* 143/15	−	−
*Escherichia coli* 131/15	−	−
*Escherichia coli* 116/15	−	−
*Escherichia coli* 90/15	−	−
*Shigella flexneri* 12022	−	−
*Salmonella enteric*a (Anatum)	−	−
*Salmonella enterica* (Heidelberg)	−	−
*Salmonella enterica* (Reading)	−	−
*Salmonella enterica* (Panama)	−	−
*Bacillus* sp.	−	−
*Pseudomonas aeruginosa* O2221	−	−
*Pseudomonas aeruginosa* O919	−	−
*Enterococcus faecalis* 271	−	−
*Enterococcus faecalis* 272	−	−
*Enterococcus faecium* 256	−	−
*Enterococcus faecium* 257	−	−
*Staphylococcus aureus* 258	−	−
*Staphylococcus aureus* 259	−	−
*Klebsiella* sp.	−	−
*Acinetobacter* sp.	−	−

Symbols: (++) regular clear plaques, (+) turbid plaques, (−) no plaques.

### Morphology and properties of vB_Eco4M-7 and ECML-117 virions and plaques

**Figure 4:**
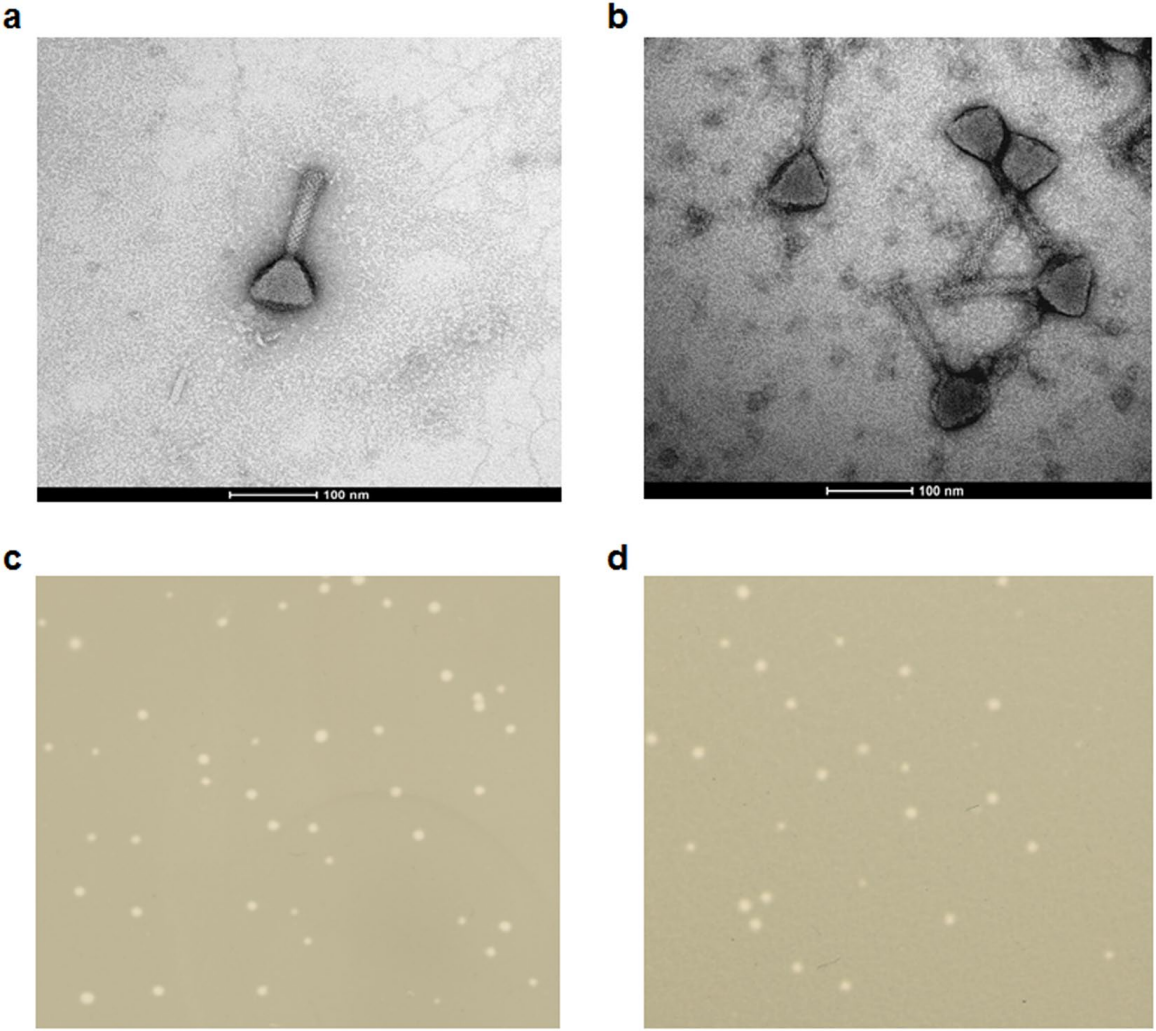
Virions and plaque morphology of *E. coli* O157:H7 phages ECML-117 and vB_Eco4M-7. Panels (**a**,**b**) present the transmission electron micrographs of phages ECML-117 (**a**) and vB_Eco4M-7 (**b**) stained with uranyl acetate (bars represent 100 nm). In the lower panels, the plaque morphologies of the tested phages ECML-117 (**c**) and vB_Eco4M-7 (**d**) examined with the double-layer agar plate method are shown.

Electron microscopic analyses indicated that vB_Eco4M-7 and ECML-117 virions consist of a head (66 nm diameter for both phages) and a contractile tail (107 ×20 nm and 120 ×20 nm for vB_Eco4M-7 and ECML-117, respectively) (Fig. 4a,b). Therefore, both phages belong to the *Myoviridae* family.

Both vB_Eco4M-7 and ECML-117 form uniform plaques on the *E. coli* O157:H7 (ST2–8624) strain, with diameters of approximately 1 mm (Fig. 4c,d). This plaque morphology is characteristic of lytic rather than temperate phages, corroborating the results of genomic analyses that suggested that vB_Eco4M-7 is a virulent phage. Again, this feature is advantageous when considering this phage for phage therapy and/or food protection.

The results of basic analyses of the sensitivity of vB_Eco4M-7 to various environmental conditions, including temperature and pH, were reported previously^14^. Here, we analysed the sensitivity of vB_Eco4M-7 and ECML-117 virions to various laboratory disinfectants. Both phages showed complete resistance to 10% soap and dish soap, Line-Antibacterial 70, and Virusolve, while being sensitive to 63% ethanol, 0.5% Virkon and 5% Viruton Pulver (Table 4).

**Table 4:** Resistance of bacteriophages ECML-117 and vB_Eco4M-7 to laboratory disinfectants. The percentage of surviving phage under certain conditions is shown.

Phage name	Percent of surviving phages						
	10% soap (2 min, RT)	10% dish soap (5 min, RT)	63% ethanol (1 h, RT)	Line-Antibacterial 70 (5 min, RT)	0.5% Virkon (30 min, RT)	Virusolve (5 min, RT)	5% Viruton Pulver (30 min, 30 °C)
ECML-117	100	100	0	100	0	100	1
vB_Eco4M-7	100	100	0.01	100	0	100	0.02

### Phage adsorption and lytic development

**Figure 5:**
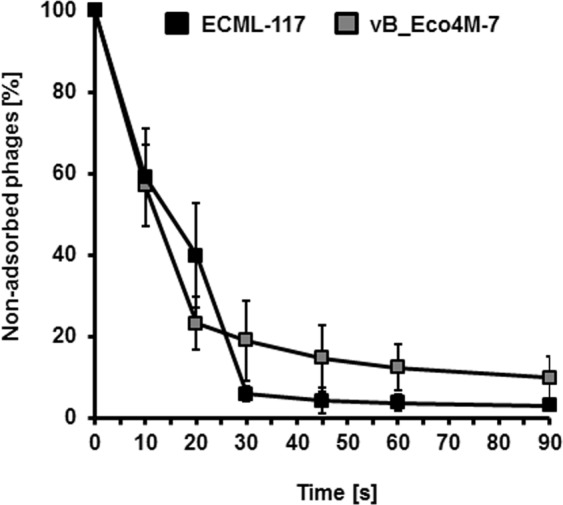
The rate of adsorption of phages ECML-117 and vB_Eco4M-7 to the *E. coli* O157:H7 (ST2–8624) host. Phage ECML-117 (black squares) or vB_Eco4M-7 (grey squares) was added to the bacterial suspension at an m.o.i. = 0.1. The percentage of nonadsorbed virions was calculated at the indicated times. The presented results are mean values from three independent experiments with SD indicated by error bars.

**Figure 6:**
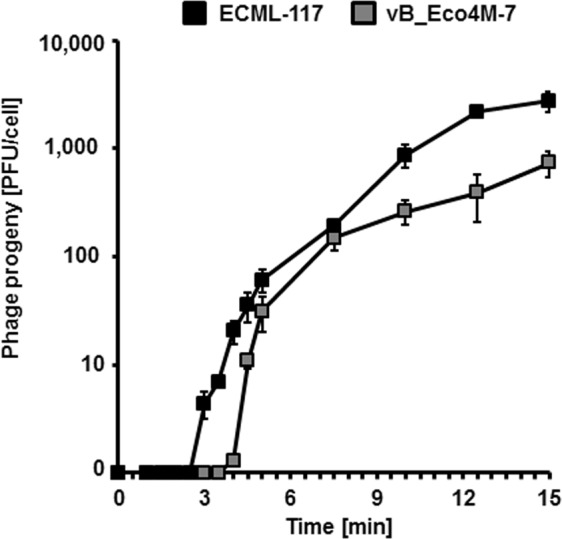
One-step growth curves of ECML-117 (black squares) and vB_Eco4M-7 (grey squares) on *E. coli* O157:H7 (ST2–8624). The results are presented as the mean values ± SD from three independent experiments.

We found that both phages, vB_Eco4M-7 and ECML-117, adsorbed efficiently on the host *E. coli* O157:H7 (ST2–8624) (Fig. 5). The adsorption was complete within approximately 1 min, and the efficiency was over 90% for both phages. Lytic development of phages vB_Eco4M-7 and ECML-117 was investigated in one-step growth experiments. These experiments indicated short eclipse and latent periods of both phages (Fig. 6). In LB medium at 37 °C, the development appeared to be complete within as short a time as 10 min, with a burst size of approximately 100 phage per cell. A further increase in phage progeny (indicated as plaque forming units per cell, pfu/cell), as shown in Fig. 6, most likely represents the second round of phage propagation after infection of host cells by phages, which appeared as progeny of initially infecting viruses.

**Figure 7:**
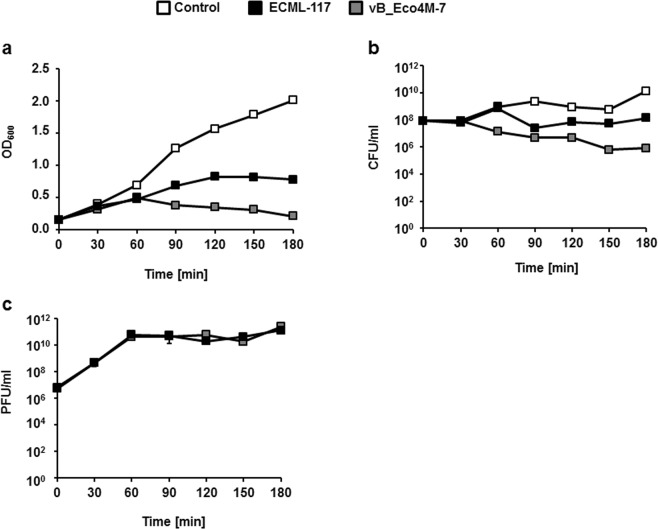
Kinetics of lytic development of phages ECML-117 (black squares) and vB_Eco4M-7 (grey squares) after infection of *E. coli* O157:H7 (ST2–8624) bacterial cells at an m.o.i. = 0.1. The results are presented as the bacterial culture density measured at OD_600_ (**a**), the number of bacterial survivors after phage infection per 1 ml (CFU/ml) (**b**), and the number of phage per 1 ml (PFU/ml) (**c**). As a negative control (white squares), the *E. coli* O157:H7 (ST2–8624) host was inoculated with LB medium instead of the tested virus. The results are presented as the mean values ± SD from three independent experiments. Note that in most cases, the error bars are smaller than the sizes of the symbols; thus, they are not visible.

When monitoring cultures of infected bacteria, a strong inhibition of bacterial growth was evident; however, vB_Eco4M-7 appeared more efficient in killing *E. coli* O157:H7 (ST2–8624) cells than ECML-117 (Fig. 7). When the number of PFU per ml of culture was estimated, the average burst size of 100 phage per cell could be confirmed, assuming that two cycles of phage propagation occurred during the tested period (Fig. 7).

The genome of *E. coli* O157:H7 (ST2–8634) contains the Shiga toxin-converting prophage ST2–8624. Lytic infection of this host by vB_Eco4M-7 did not induce ST2–8624 (Supplementary Fig. S1). After one-step growth experiments and lysis profile assays with the tested phage and host bacterium containing the prophage ST2–8624, no plaques appeared when lysates were spotted onto agar plates with the *E. coli* C600 strain, indicating that the prophage was not induced (Supplementary Figs. S1a and S1b). Moreover, in the *E. coli* O157:H7 host bearing the ST2–8624 prophage, which contained the *gfp* gene instead of the *stx* genes, no increase in the amount of GFP could be observed, contrary to control experiments with mitomycin C-mediated prophage induction (Supplementary Fig. S1c).

The kinetics of lytic development of the vB_Eco4M-7 and ECML-117 phages indicated their properties, which are promising for their use in phage therapy and/or food protection, i.e., rapid lytic development, efficient killing of host bacteria, and sufficiently high burst size.

## Conclusions

The results of the studies presented in this report indicate that the vB_Eco4M-7 and ECML-117 viruses can be considered promising phages in further studies on their use in phage therapy and/or food protection. Specifically, the following properties are especially advantageous: (I) an apparent lack of genes coding for toxins (suggested on the basis of genome analysis against the Virulence Factors of Pathogenic Bacteria database; VFDB); (II) a lack of lysogenization-specific genes and formation of clear plaques, indicating the virulence-only type of development; (III) effective infection of many, though not all, *E. coli* O157 strains; (IV) efficient adsorption to host cells; (V) rapid (eclipse time as short as 3–5 min) and efficient (average burst size approximately 100 phage per cell) lytic development; and (VI) relatively high resistance to various environmental conditions and different disinfectants. Therefore, we suggest that further works towards applications of vB_Eco4M-7 and ECML-117 in medicine and/or food protection are substantiated.

## Methods

### Bacterial strains and culture conditions

The bacterial strains used in this study are listed in Supplementary Table S4. For all experiments, the tested bacteria were cultivated in LB (*E. coli*, *Salmonella*, *Bacillus*, *Pseudomonas*, *Staphylococcus*, *Klebsiella* and *Acinetobacter* strains) or TSB (*Enterococcus* and *Shigellas* strains) liquid medium at 37 °C on a rotary shaker at a rate of 200 rpm. LB with 1.5% agar or TSA were used as solid media. Plates with bacterial strains were incubated overnight at 37 °C.

### Isolation and propagation of bacterial viruses

Two bacteriophages, ECML-117 and vB_Eco4M-7, were employed in this study. ECML-117 (ATCC PTA-7950; GenBank: JX128258.1) was obtained from ATCC: The Global Bioresource Center. The second phage, vB_Eco4M-7, was isolated from urban sewage collected from the Gdansk Wastewater Treatment Plant in Poland, as described previously^14^. To obtain lysates, *E. coli* O157:H7 (ST2–8624) at the early log phase (OD_600_ = 0.2) was infected by ECML-117 or vB_Eco4M-7 at 37 °C and cultivated for 2 h with shaking. Cell debris was removed by centrifugation (2,000 g, 10 min, 4 °C). The supernatant was passed through 0.22-µm-pore-size filters, yielding a crude extract of phage particles. To determine the number of phage per ml (PFU/ml), the suspensions of ECML-117 and vB_Eco4M-7 were diluted 10-fold in TM buffer (10 mM Tris-HCl, 10 mM MgSO_4_; pH 7.2) and tested for plaque formation with *E. coli* O157:H7 (ST2–8624) by using the double-layer agar plate method. Plaques were observed on the plates after 20 h of incubation at 37 °C.

### Host range analysis

The ability of ECML-117 and vB_Eco4M-7 to infect different bacterial strains was tested. In the first step, 1 ml of potential host bacteria was mixed with 2 ml of melted agar (LB with 0.7% agar or TSB with 0.4% agarose) and poured onto solid agar (LB with 1.5% agar or TSA) to make double-layer plates. Then, the phage suspension was diluted in TM buffer (10 mM Tris-HCl, 10 mM MgSO_4_; pH 7.2) and spotted onto plates containing the tested bacterial strain. After overnight incubation at 37 °C, the interaction of the bacterial virus with the host was confirmed by the presence of clear zones at the sites of phage application. The areas that looked no different from the surrounding untreated lawn were scored as negative. According to the plaque morphology, the obtained results were differentiated into three groups: (++) regular clear plaques, (+) turbid plaques, and (−) no plaques.

### Electron microscopy

Purification of ECML-117 and vB_Eco4M-7 particles was carried out by using the caesium chloride density gradient centrifugation method, according to the procedure described previously^15^. Transmission electron microscopy analysis of the phages was performed in the Laboratory of Electron Microscopy, Faculty of Biology, University of Gdansk, Gdansk, Poland. Virions were negatively stained with uranyl acetate, and then, micrographs were taken under a Philips CM 100 electron microscope (Philips, Eindhoven, The Netherlands).

### Plaque morphology

The plaque morphology of the ECML-117 and vB_Eco4M-7 bacteriophages was tested on the *E. coli* O157:H7 (ST2–8624) strain. To determine the plaque size, 10-fold dilutions of virus stocks were prepared in TM buffer (10 mM Tris-HCl, 10 mM MgSO_4_; pH 7.2). In the next step, 1 ml of overnight host culture was mixed with 25 µl of an appropriate dilution of phage lysate and added to 2 ml of LB with 0.7% agar. The mixture was poured onto plates containing 25 ml of LB agar. The double-layer agar plates were incubated at 37 °C for 20 h. The next day, plaque morphology and diameter were determined.

### The influence of laboratory disinfectants on phage viability

The stability of the phage particles was tested against seven disinfectants (10% dish soap, 10% hand soap, 63% ethanol, Line Antibacterial 70, 0.5% VirkonS, Virusolve and 5% Viruton Pulver) used in the laboratory. All experiments were prepared at room temperature according to the protocol supplied from the provider. The phage suspensions were mixed with the tested disinfectants at a 1:9 ratio, and after incubation, the lysate titer was determined by the double-layer method. Bacterial viruses incubated in TM buffer (10 mM Tris-HCl, 10 mM MgSO_4_; pH 7.2) without disinfectants were used as controls. After overnight incubation at 37 °C, the percentage of remaining phage particles able to form plaques was calculated.

### Phage adsorption to bacterial host cells

To determine the kinetics of phage adsorption to the *E. coli* O157:H7 (ST2–8624) host, 10^9^ cells were infected with a phage suspension to reach an m.o.i. = 0.1 and then incubated at 37 °C. After 0, 10, 20, 30, 40, 50, 60, and 90 s, three individual samples per phage were collected and centrifuged at 6,000 g for 1 min to sediment the bacterial cells with the adsorbed phages. The supernatant was diluted in TM buffer (10 mM Tris-HCl, 10 mM MgSO_4_; pH 7.2) and assayed for free, unadsorbed phage particles. The number of viruses mixed with bacterial host cells at time 0 was considered 100% nonadsorbed phages. Other values were compared to this sample.

### One-step growth assay

One-step growth experiments were performed as described previously^16^, with some modifications. In brief, *E. coli* O157:H7 (ST2–8624) was grown until the early exponential phase (2 × 10^8^ CFU/ml). Then, 10 ml of bacterial culture was centrifuged (4000 × g, 10 min, 4 °C), and the pellet was suspended in 1 ml of LB medium supplemented with 3 mM sodium azide. In the next step, phage particles were added to the host at an m.o.i. = 0.1 and allowed to adsorb for 10 min at 37 °C. The mixture was centrifuged at 4,000 × g for 10 min to remove unadsorbed viruses. After three washes, the bacterial pellet was resuspended in LB medium containing 3 mM sodium azide; then, 25 µl of the bacterial mixture was added to 25 ml of LB medium (time 0) and cultivated at 37 °C. The number of infective centres was estimated from samples taken 1 min after infection by plating under permissive conditions. All samples were cleared by centrifugation and titrated to determine the number of PFU per ml. The plates were incubated at 37 °C overnight. Burst size was calculated as the ratio of phage titer to the number of infection centres.

### Lysis profile of host bacteria after phage infection

*E. coli* O157:H7 (ST2–8624) cells were cultivated to OD_600_ = 0.15 at 37 °C. Then, phage stock solution was added to the host at an m.o.i. = 0.1. Bacterial growth was monitored by measuring the OD_600_ at various time points. As a negative control, host bacteria were inoculated with LB medium instead of the tested phage. The bacterial density was recorded at 30 min intervals over the period of 180 min. During this experiment, the number of bacterial cells per ml (CFU/ml) and phage titer (PFU/ml) were also determined. To calculate the number of surviving cells after virus infection, 100 µl of bacterial culture was collected at the indicated times and diluted 10-fold in 0.85% sodium chloride. In the next step, 40 µl of each dilution was spread onto LB agar plates. The CFU/ml was calculated on the basis of the counted colonies after overnight incubation at 37 °C. To estimate the PFU/ml, samples were taken every 30 min, and after dilution in TM buffer (10 mM Tris-HCl, 10 mM MgSO_4_; pH 7.2), the mixture was spotted onto a double-layer agar plate. The phage titer was determined by counting single plaques.

### The influence of vB_Eco4M-7 or ECML-117 infection on ST2–8624 prophage induction in the lysogenic host

To test whether the infection of *E. coli* O157:H7 (ST2–8624) bacteria with lytic phages causes the induction of prophage ST2–8624, the lysis profile and one-step growth assays were performed according the procedures described above. During these experiments, the titer of phage ST2–8624 (PFU/ml) was tested. The samples were collected at the indicated times, and after dilution in TM buffer (10 mM Tris-HCl, 10 mM MgSO_4_; pH 7.2), the mixture was spotted onto a double-layer agar plate containing the *E. coli* C600 indicator strain. The PFU/ml was determined by counting single plaques.

In the ST2–8624 (Δ*stx::cat gfp)* genome, the *stx* locus was replaced with the *cat* and *gfp* genes. Thus, the efficiency of expression of the *gfp* gene corresponds to that of the *stx* gene in the wild-type phage. For estimation of the level of GFP protein in bacteria bearing the ST2–8624 prophage during infection with vB_Eco4M-7 or ECML-117, 150 µL of the culture was harvested and transferred to a 96-well polystyrene plate at each time point (each sample was analysed in triplicate). GFP fluorescence (excitation at 395 nm, emission at 509 nm) was measured for 1 s in the EnSpire Multimode Plate Reader. As a positive control, host bacteria were inoculated with LB medium instead of phage vB_Eco4M-7 or ECML-117 and treated with mitomycin C at a final concentration of 1 µg/ml. The negative control was the bacterial culture without an induction agent and the tested lytic bacteriophages.

### Isolation and sequencing of the vB_Eco4M-7 genome

The purified phage sample was treated with DNaseI and RNaseA for 30 min at 37 °C to digest the exogenous bacterial RNA and DNA. After thermal inactivation, the suspension was treated with proteinase K for 60 min at 37 °C. The phage genome DNA was isolated by using the MasterPure™ Complete DNA and RNA Purification Kit (Epicentre, Madison, U.S.A.) according to the procedure described in the manual. The DNA concentration was determined spectrophotometrically by measuring the absorbance at a wavelength of 260 nm. The vB_Eco4M-7 genomic DNA was sequenced by the Genomed company with Next Generation Sequencing (NGS) and MiSeq (Illumina) genome sequencing. The quality of the vB_Eco4M-7 reads was verified using FastQC (https://www.bioinformatics.babraham.ac.uk/projects/fastqc/). To remove the adapters, N bases, and low-quality reads, the raw data (400,818 raw reads) were filtered using the Cutadapt program (http://code.google.com/p/cutadapt/) with the following parameters: −q = 20 and −m = 36. De novo assembly with 392,358 trimmed reads (97.88% of raw reads) was performed using CLC Genomics Workbench. Finally, a single contig, corresponding to the entire phage vB_Eco4M-7 with an average coverage of 1,130 ×, was generated.

### Annotation and bioinformatic analysis of the vB_Eco4M-7 genome

Putative open reading frames (ORFs) were predicted by using myRAST software^17^ and UGENE bioinformatics software (http://ugene.net/)^18^. The analysis of the putative protein-coding genes was based on the presence of the ribosomal binding site and start and stop codons. Prepared annotations were also verified by BLAST analysis, HMMER software (http://www.ebi.ac.uk/Tools/hmmer/), Phobious webserver (http://phobius.binf.ku.dk/) and the TMHMM program (http://www.cbs.dtu.dk/services/TMHMM/). The putative functions of translated products were analysed and annotated using BLASTp and PHASTER Prophage/Virus databases^19^. The NCBI Conserved Domain Database was also used for prediction of evolutionarily conserved protein domains and motifs^20^. ShortBRED was used to search the virulence factors and toxins in predicted ORFs against the Virulence Factors of Pathogenic Bacteria database (VFDB)^21,22^. The BLAST Ring Image Generator (BRIG) platform (https://sourceforge.net/projects/brig/) was used to create a circular map of the vB_Eco4M-7 genome. To perform GC skew and GC content analyses^23^, CGView was employed. Comparison of ORFs from relative phages, vB_EcoM_WFC (MK373777.1), ECML-117 (JX128258.1), vB_EcoM_WFH (MK373776.1), FEC19 (MH816966.1) and vB_EcoM-Ro157lw (MH051335.1), was performed by using the EasyFig program (http://mjsull.github.io/Easyfig/files.html). The phage-specific promoters and the Rho-independent transcriptional terminators in viral DNA sequences were predicted by the Neural Network Promoter Prediction NNPP method (http://www.fruitfly.org/seq_tools/promoter.html) and FindTerm tool (http://www.softberry.com/berry.phtml), respectively. The genome sequence of vB_Eco4M-7 was deposited in GenBank under the accession number MN176217.

### Phylogenetic analysis

Phylogenetic analyses between the genome of vB_Eco4M-7 and the genomes of related phages were performed using MUSCLE from MEGA software (http://www.megasoftware.net/). To construct the phylogenetic tree, the amino acid sequence of the terminase large subunit (genetic marker for the order *Caudovirales)* of the vB_Eco4M-7 virus was compared with the sequences of other reference bacteriophages within the order *Caudovirales* that were deposited in the NCBI database. The neighbour-joining phylogenetic tree was constructed using the Poisson model, and the robustness of the tree topology was assessed by bootstrap analyses based on 1 000 replicates.

### Extraction of phage proteins

The analyses of ECML-117 and vB_Eco4M-7 proteins were carried out at the Institute of Bioorganic Chemistry of Polish Academy of Sciences. In the first step, the phage lysate obtained after caesium chloride density gradient centrifugation was incubated with ice-cold acetone at −20 °C for 30 min. After centrifugation, the pellet was suspended in 50 mM ammonium bicarbonate, and the total concentration of bacterial virus proteins was estimated by using a BCA colorimetric assay according to the procedure described in the protocol. Then, an appropriate aliquot of phage protein lysate was treated with 5.6 mM dithiothreitol (DTT) in 50 mM ammonium bicarbonate and incubated at 95 °C for 5 min. The sample was cooled to room temperature, alkylated with 5 mM iodoacetamide and kept for 20 min in the dark. In the next step, phage proteins were digested with 0.2 µg of sequencing-grade trypsin. After overnight incubation at 37 °C, the enzyme was inactivated by adding trifluoroacetic acid (TFA), and the mixture was transferred to an HPLC conical vial.

### Mass spectrometry analysis of viral proteins

The analysis of phage proteins was performed by employing the Dionex UltiMate 3000 RSLC nanoLC System connected to a Q Exactive Orbitrap mass spectrometer (Thermo Fisher Scientific). In the first step, peptides obtained after trypsin digestion were separated on a reverse phase Acclaim PepMap RSLC nanoViper C18 column by using an acetonitrile gradient. Mass spectra were acquired on the Q Exactive instrument in a data-dependent mode by using the top 10 data-dependent MS/MS scans. The target value for the full scan MS spectra was set to 1e6 with a maximum injection time of 100 ms and a resolution of 70,000 at m/z 400. The 10 most intense ions charged two or more were selected with an isolation window of 2 Da and fragmented by higher energy collisional dissociation with NCE 27. The ion target value for MS/MS was set to 5e4 with a maximum injection time of 100 ms and a resolution of 17,500 at m/z 400. Identification of bacterial virus proteins was performed by using Proteome Discoverer 1.4 software (Thermo Scientific) and a database created from amino acid sequences of proteins that were encoded by genes in the genomes of ECML-117 or vB_Eco4M-7. Proteins were classified as positively identified if at least two peptide spectral matches per protein were found by the Sequest search engine, and a peptide score reached the significance threshold when FDR = 0.05.

## Supplementary information

Related Manuscript File.

Supplementary Table S1.

Supplementary Table S2.

Supplementary Table S3.

## References

[1] Vivas R, Barbosa AAT, Dolabela SS, Jain S (2019). Multidrug-resistant bacteria and alternative methods to control them: an overview. Microb. Drug. Resist..

[2] Kutter EM, Kuhl SJ, Abedon ST (2015). Re-establishing a place for phage therapy in western medicine. Future Microbiol..

[3] Domingo-Calap P, Delgado-Martínez J (2018). Bacteriophages: protagonists of a post-antibiotic era. Antibiotics.

[4] Górski A (2018). Phage therapy: what have we learned?. Viruses.

[5] Górski, A. *et al*. Phage therapy: current status and perspectives. *Med. Res. Rev*, 1–5; 10.1002/med.21593 (2019). 10.1002/med.21593 31062882

[6] Kakasis A, Panitsa G (2019). Bacteriophage therapy as an alternative treatment for human infections. A comprehensive review. Int. J. Antimicrob. Agents.

[7] Gutiérrez D, Rodríguez-Rubio L, Martínez B, Rodríguez A, García P (2016). Bacteriophages as weapons against bacterial biofilms in the food industry. Front. Microbiol..

[8] Gyles CL (2007). Shiga toxin-producing *Escherichia coli*: an overview. J. Anim. Sci..

[9] Nataro JP, Kaper JB (1998). Diarrheagenic *Escherichia coli*. Clin. Microbiol. Rev..

[10] Sandvig K, van Deurs B (1996). Endocytosis, intracellular transport, and cytotoxic action of Shiga toxin and ricin. Physiol. Rev..

[11] Muniesa M, Hammerl JA, Hertwig S, Appel B, Brüssow H (2012). Shiga toxin-producing *Escherichia coli* O104:H4: a new challenge for microbiology. Appl. Environ. Microbiol..

[12] Bloch SK (2012). *Escherichia coli* O104:H4 outbreak- have we learnt a lesson from it?. Acta Biochim. Pol..

[13] Łoś JM, Łoś M, Węgrzyn G (2011). Bacteriophages carrying Shiga toxin genes: genomic variations, detection and potential treatment of pathogenic bacteria. Future Microbiol..

[14] Jurczak-Kurek A (2016). Biodiversity of bacteriophages: morphological and biological properties of a large group of phages isolated from urban sewage. Sci. Rep..

[15] Sambrook, J. & Russell, D. W. *Molecular Cloning: A Laboratory Manual*, 3rd ed. (NY: Cold Spring Harbor Laboratory Press, 2001).

[16] Bloch S (2013). Genes from the exo-xis region of λ and Shiga toxin-converting bacteriophages influence lysogenization and prophage induction. Arch. Microbiol..

[17] Caldeira JC, Peabody DS (2007). Stability and assembly *in vitro* of bacteriophage PP7 virus-like particles. J. Nanobiotechnol..

[18] Essoh C (2015). Investigation of a large collection of *Pseudomonas aeruginosa* bacteriophages collected from a single environmental source in Abidjan, Côte d’Ivoire. PLoS ONE.

[19] Arndt D (2016). Phaster: A better, faster version of the phast phage search tool. Nucleic Acids Res..

[20] Marchler-Bauer A (2017). CDD/SPARCLE: functional classification of proteins via subfamily domain architectures. Nucleic Acids Res..

[21] Philipson CW (2018). Characterizing Phage Genomes for Therapeutic Applications. Viruses..

[22] Chen L, Zheng D, Liu B, Yang J, Jin Q (2016). VFDB 2016: Hierarchical and refined dataset for big data analysis—10 years on. Nucleic Acids Res..

[23] Stothard P, Wishart DS (2005). Circular genome visualization and exploration using CGView. Bioinformatics.

