# Supplementary material for: Characterization of a bacteriophage, vB_Eco4M-7, that effectively infects many Escherichia coli O157 strains
Source: Sci Rep. 2020 Feb 28;10:3743. doi: 10.1038/s41598-020-60568-4 (PMC7048862; doi:10.1038/s41598-020-60568-4)
Supplement: Supplementary file 1 — Related Manuscript File. [file 41598_2020_60568_MOESM1_ESM.docx]

**Supplementary information**

**Characterization of a bacteriophage, vB_Eco4M-7, that effectively infects many *Escherichia coli* O157 strains**

**Agnieszka Necel^1^, Sylwia Bloch^2^, Bożena Nejman-Faleńczyk^1^, Michał Grabski^1^, Gracja Topka^1^, Aleksandra Dydecka^1^, Katarzyna Kosznik-Kwaśnicka^2^, Łukasz Grabowski^2^, Agata Jurczak-Kurek^3^, Tomasz Wołkowicz^4^, Grzegorz Węgrzyn^1^, Alicja Węgrzyn^2,^***

^1^ *Department of Molecular Biology, Faculty of Biology, University of Gdańsk, Wita Stwosza 59, 80-308 Gdańsk, Poland*

^2^ *Laboratory of Molecular Biology, Institute of Biochemistry and Biophysics, Polish Academy of Sciences, Kładki 24, 80-822 Gdańsk, Poland*

^3^ *Department of Molecular Evolution, Faculty of Biology, University of Gdańsk, Wita Stwosza 59, 80-308 Gdańsk, Poland*

^4^ *Department of Bacteriology and Biocontamination Control, National Institute of Public Health-National Institute of Hygiene, Chocimska 24, 00-791 Warsaw, Poland.*


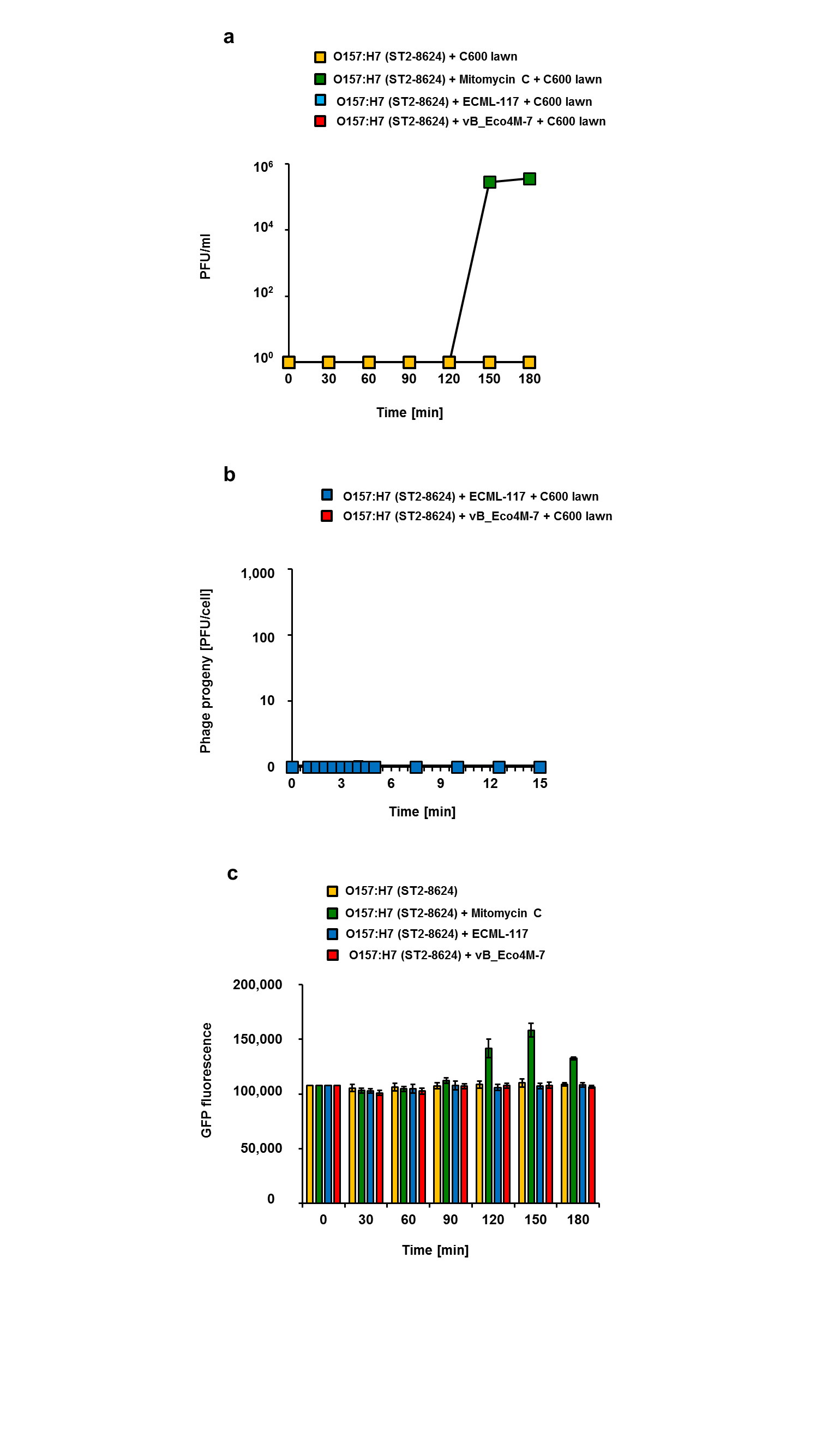


**Supplementary Figure S1.** The development of ST2-8624 prophage after infection of host bacteria with ECML-117 (blue squares) or Eco4M-7 (red squares). Results are presented as a number of phages per 1 ml (PFU/ml) (a), as a number of phages (PFU) per cell (the burst size; PFU/cell) (b), and the level of GFP fluorescence in bacterial cultures (c). As a negative control (yellow squares), *E. coli* O157:H7 (ST2-8624) host was inoculated with LB medium instead of tested virus. As a positive control (green squares), *E. coli* O157:H7 (ST2-8624) host was inoculated with LB medium instead of tested virus and treated with 1 µg/ml of mitomycin C. Results are presented as mean values ± SD from three independent experiments.

**Supplementary Table S4.** Bacterial strains

| **Bacterial strain** | **Source or references** | **Genotype or other characteristics** |
| --- | --- | --- |
| ***E. coli* laboratory strains** | | |
| C600 | 1 | F- *tonA21 thi-1 thr-1 leuB6 lacY1 glnV44 rfbC1 fhuA1* λ- |
| DH5α | 2 | F- *endA1 glnV44 thi-1 recA1 gyrA96 deoR nupG Ф80dlacZΔM15 Δ(lacZYA-argF)U169, hsdR17(rK- mK+),* λ*-* |
| Hfr300 | 3 | Hfr |
| MG1655 | 4 | F- l– *ilvG rfb-50 rph*-1 |
| MC1061 | 5 | FΔ*(ara-leu)7697 [araD139]B/r* Δ*(codB-lacI)3 galK16 galE15 λ- e14- mcrA0 relA1 rpsL150(strR) spoT1 mcrB1 hsdR2(r-m+)* |
| Tap90 | 6 | F- *recD1903::mini-tet*  *supE44 supF58 lacY1 pro leuB6 hsdR rpsL tonA1 thi-1* λ - |
| ***E. coli* clinical strains** | | |
| EPEC-A | Specialist Hospital of St. Wojciech in Gdansk | stool isolate; EspA |
| EPEC-B | Specialist Hospital of St. Wojciech in Gdansk | stool isolate; EspB |
| EPEC-C | Specialist Hospital of St. Wojciech in Gdansk | stool isolate; EspC |
| O157:H7 700728 | ATCC: The Global Bioresource Center | - |
|  |  |  |
| O157:H7 17076 | DSMZ – German Collection of Microorganisms and Cell Cultures GmbH | stool isolate, non-STEC |
|  |  |  |
| O157:H7 19206 | DSMZ – German Collection of Microorganisms and Cell Cultures GmbH | stool isolate, non-STEC |
| O157:H7 (ST2-8624) | 7 | stool isolate; Stx1 and Stx2 |
| O157:H7 (ST2-8624) | 8 | Δ*stx::cat gfp* |
| O157:H7 EDL933 | 9 & 10 | stool isolate; Stx1 and Stx2 |
| O157:H7 CB571 | 9 | stool isolate; Stx1 and Stx2 |
| O157:H7 174/03 | National Institute of Public Health-Public Institute of Hygiene  11 | stool isolate; Stx2 |
| O157:H7 598/03 | National Institute of Public Health-Public Institute of Hygiene  11 | stool isolate; Stx1and Stx2 |
| O157:H7 49/04 | National Institute of Public Health-Public Institute of Hygiene  11 | stool isolate; Stx1and Stx2 |
| O157:H7 365/05 | National Institute of Public Health-Public Institute of Hygiene  11 | stool isolate; Stx1and Stx2 |
| O157:H7 175/06 | National Institute of Public Health-Public Institute of Hygiene  11 | stool isolate; Stx1and Stx2 |
| O157:H7 206/06 | National Institute of Public Health-Public Institute of Hygiene  11 | stool isolate; Stx1and Stx2 |
| O157:H7 474/07 | National Institute of Public Health-Public Institute of Hygiene  11 | stool isolate; Stx1and Stx2 |
| O157:H7 371/08 | National Institute of Public Health-Public Institute of Hygiene  11 | stool isolate; Stx2 |
| O157:H7 4/10 | National Institute of Public Health-Public Institute of Hygiene  11 | stool isolate; Stx1and Stx2 |
| O157:H7 251/10 | National Institute of Public Health-Public Institute of Hygiene  11 | stool isolate; Stx1and Stx2 |
| O157:H7 79/13 | National Institute of Public Health-Public Institute of Hygiene | stool isolate; Stx1and Stx2 |
| O157:H7 242/13 | National Institute of Public Health-Public Institute of Hygiene | stool isolate; Stx1 and Stx2 |
| O157:H7 262/13 | National Institute of Public Health-Public Institute of Hygiene | stool isolate; Stx1and Stx2 |
| O157:H7 224/14 | National Institute of Public Health-Public Institute of Hygiene | stool isolate; Stx1and Stx2 |
| O157:H7 226/14 | National Institute of Public Health-Public Institute of Hygiene | stool isolate; Stx2 |
| O157:H7 58/17 | National Institute of Public Health-Public Institute of Hygiene | stool isolate; Stx1and Stx2 |
| O157:H7 8185 | National Institute of Public Health-Public Institute of Hygiene | Stx2 |
| O157 225/96 | National Institute of Public Health-Public Institute of Hygiene  11 | stool isolate, Stx1and Stx2 |
| O157 440/98 | National Institute of Public Health-Public Institute of Hygiene  11 | stool isolate, Stx2 |
| O157 19/99 | National Institute of Public Health-Public Institute of Hygiene  11 | stool isolate, Stx1and Stx2 |
| O157 568/99 | National Institute of Public Health-Public Institute of Hygiene  11 | stool isolate, Stx1and Stx2 |
| O157 286/00 | National Institute of Public Health-Public Institute of Hygiene  11 | stool isolate, Stx2 |
| O157 214/04 | National Institute of Public Health-Public Institute of Hygiene  11 | stool isolate, Stx1 and Stx2 |
| O157 443/07 | National Institute of Public Health-Public Institute of Hygiene  11 | stool isolate, Stx1and Stx2 |
| O157 131/17 | National Institute of Public Health-Public Institute of Hygiene | stool isolate, Stx2 |
| O157 99/18 | National Institute of Public Health-Public Institute of Hygiene | stool isolate, Stx2 |
| O157 4/19 | National Institute of Public Health-Public Institute of Hygiene | stool isolate, Stx1and Stx2 |
| O157 345/96 | National Institute of Public Health-Public Institute of Hygiene  11 | food isolate, Stx2 |
| O157 346/96 | National Institute of Public Health-Public Institute of Hygiene  11 | food isolate, Stx2 |
| O157 347/96 | National Institute of Public Health-Public Institute of Hygiene  11 | food isolate, Stx2 |
| O157 42/16 | National Institute of Public Health-Public Institute of Hygiene | stool isolate, non-STEC |
| O157 95/16 | National Institute of Public Health-Public Institute of Hygiene | stool isolate, non-STEC |
| O157 99/16 | National Institute of Public Health-Public Institute of Hygiene | stool isolate, non-STEC |
| O157 156/16 | National Institute of Public Health-Public Institute of Hygiene | stool isolate, non-STEC |
| O157 18/19 | National Institute of Public Health-Public Institute of Hygiene | stool isolate, non-STEC |
| O157 13/19 | National Institute of Public Health-Public Institute of Hygiene | stool isolate, non-STEC |
| O25 191/19 | National Institute of Public Health-Public Institute of Hygiene | stool isolate, non-STEC |
| O25 49/19 | National Institute of Public Health-Public Institute of Hygiene | stool isolate, non-STEC |
| O25 13/19 | National Institute of Public Health-Public Institute of Hygiene | stool isolate, non-STEC |
| O25 171/18 | National Institute of Public Health-Public Institute of Hygiene | stool isolate, non-STEC |
| O25 170/18 | National Institute of Public Health-Public Institute of Hygiene | stool isolate, non-STEC |
| O25 169/18 | National Institute of Public Health-Public Institute of Hygiene | stool isolate, non-STEC |
| O25 84/17 | National Institute of Public Health-Public Institute of Hygiene | stool isolate, non-STEC |
| O25 45/16 | National Institute of Public Health-Public Institute of Hygiene | stool isolate, non-STEC |
|  |  |  |
| O25 43/16 | National Institute of Public Health-Public Institute of Hygiene | stool isolate, non-STEC |
| O25 191/15 | National Institute of Public Health-Public Institute of Hygiene | stool isolate, non-STEC |
| O26 113/19 | National Institute of Public Health-Public Institute of Hygiene | stool isolate, Stx2 |
| O26 214/15 | National Institute of Public Health-Public Institute of Hygiene | stool isolate, non-STEC |
| O44 254/15 | National Institute of Public Health-Public Institute of Hygiene | stool isolate, non-STEC |
| O44 166/15 | National Institute of Public Health-Public Institute of Hygiene | stool isolate, non-STEC |
| O55 22/17 | National Institute of Public Health-Public Institute of Hygiene | stool isolate, non-STEC |
| O86 149/17 | National Institute of Public Health-Public Institute of Hygiene | stool isolate, non-STEC |
| O111 89/15 | National Institute of Public Health-Public Institute of Hygiene | stool isolate, non-STEC |
| O119 137/18 | National Institute of Public Health-Public Institute of Hygiene | stool isolate, non-STEC |
| O125 162/19 | National Institute of Public Health-Public Institute of Hygiene | stool isolate, non-STEC |
| O126 10/18 | National Institute of Public Health-Public Institute of Hygiene | stool isolate, non-STEC |
| O127 60/17 | National Institute of Public Health-Public Institute of Hygiene | stool isolate, non-STEC, Eae |
| O127 53/17 | National Institute of Public Health-Public Institute of Hygiene | stool isolate, non-STEC |
| O128 145/17 | National Institute of Public Health-Public Institute of Hygiene | stool isolate, non-STEC |
| O128 55/17 | National Institute of Public Health-Public Institute of Hygiene | stool isolate, non-STEC, Eae |
| 185/19 | National Institute of Public Health-Public Institute of Hygiene | stool isolate, non-STEC |
| 135/19 | National Institute of Public Health-Public Institute of Hygiene | stool isolate, non-STEC |
| 111/19 | National Institute of Public Health-Public Institute of Hygiene | stool isolate, non-STEC |
| 16/19 | National Institute of Public Health-Public Institute of Hygiene | stool isolate, non-STEC |
| 3/19 | National Institute of Public Health-Public Institute of Hygiene | stool isolate, non-STEC |
| 93/16 | National Institute of Public Health-Public Institute of Hygiene | stool isolate, non-STEC |
| 91/16 | National Institute of Public Health-Public Institute of Hygiene | stool isolate, non-STEC |
| 90/16 | National Institute of Public Health-Public Institute of Hygiene | stool isolate, non-STEC |
| 68/16 | National Institute of Public Health-Public Institute of Hygiene | stool isolate, non-STEC |
| 65/16 | National Institute of Public Health-Public Institute of Hygiene | stool isolate, non-STEC |
| 12/16 | National Institute of Public Health-Public Institute of Hygiene | stool isolate, non-STEC |
| 296/15 | National Institute of Public Health-Public Institute of Hygiene | stool isolate, non-STEC |
| 246/15 | National Institute of Public Health-Public Institute of Hygiene | stool isolate, non-STEC |
| 146/15 | National Institute of Public Health-Public Institute of Hygiene | stool isolate, non-STEC |
| 144/15 | National Institute of Public Health-Public Institute of Hygiene | stool isolate, non-STEC |
| 143/15 | National Institute of Public Health-Public Institute of Hygiene | stool isolate, non-STEC |
| 131/15 | National Institute of Public Health-Public Institute of Hygiene | stool isolate, non-STEC |
| 116/15 | National Institute of Public Health-Public Institute of Hygiene | stool isolate, non-STEC |
| 90/15 | National Institute of Public Health-Public Institute of Hygiene | stool isolate, non-STEC |
| 3250 | Specialist Hospital of St. Wojciech in Gdansk (Poland) | stool isolate |
| 23580 | Specialist Hospital of St. Wojciech in Gdansk (Poland) | stool isolate |
| 23581 | Specialist Hospital of St. Wojciech in Gdansk (Poland) | stool isolate |
| **Other strains** |  |  |
| *Enterococcus faecalis* 271 | Hospital of St. Wincenty a Paulo in Gdynia (Poland) | urine isolate |
| *Enterococcus faecalis* 272 | Hospital of St. Wincenty a Paulo in Gdynia (Poland) | urine isolate |
| *Enterococcus faecium* 256 | Clinic of Hyperbaric Medicine and Maritime Rescue in Gdynia (Poland) | pus isolate,VRE |
| *Enterococcus faecium* 257 | Specialist Hospital in Prabuty (Poland) | pus isolate,VRE |
| *Pseudomonas aeruginosa* O919 | Hospital of St. Wincenty a Paulo in Gdynia (Poland) | bronchial mucus |
| *Pseudomonas aeruginosa* O2221 | Hospital of St. Wincenty a Paulo in Gdynia (Poland) | bronchial mucus |
| *Salmonella enterica* Anatum | National Salmonella Center at Medical University of Gdansk (Poland) | - |
| *Salmonella enterica* Heidelberg | National Salmonella Center at Medical University of Gdansk (Poland) | - |
| *Salmonella enterica* Panama | National Salmonella Center at Medical University of Gdansk (Poland) | - |
| *Salmonella enterica* Reading | National Salmonella Center at Medical University of Gdansk (Poland) | - |
| *Shigella flexnerii* 12022 | ATCC: The Global Bioreosurce Center | - |
| *Staphylococcus aureus* 258 | Hospital of St. Franciszek Żaczk in Puck (Poland) | wound isolate, MRSA |
| *Staphylococcus aureus* 259 | Hospital of St. Franciszek Żaczk in Puck (Poland) | wound isolate, MRSA |
| *Shigella flexnerii* 12022 | ATCC: The Global Bioreosurce Center | - |
| *Acinetobacter* sp. | Department of Molecular Biology at University of Gdansk | - |
| *Bacillus* sp. | Department of Molecular Biology at University of Gdansk | - |
| *Klebsiella* sp. | Department of Molecular Biology at University of Gdansk | - |

**Abbreviations:**, **EPEC** – Enteropathogenic *E. coli*, **STEC** – Shiga toxin-producing *E. coli*  **Stx** – Shiga toxin, **Esp** – *E. coli* secretion protein, **Eae** – *E. coli* intimin adherence protein, **VRE** - Vancomycin-Resistant *Enterococcus*, **MRSA** - Methicyllin-Resistant *Staphylococcus aureus*

**REFERENCES**

1. [Appleyard, R.K](https://www.ncbi.nlm.nih.gov/pubmed/?term=Appleyard%20RK%5BAuthor%5D&cauthor=true&cauthor_uid=17247495). Segregation of new lysogenic types during growth of a doubly lysogenic strain derived from *Escherichia Coli* K12. *Genetics* **39**, 440-452 (1954).
2. Taylor, R.G., Walker, D.C. & McInnes, R.R. *E. coli* host strains significantly affect the quality of small scale plasmid DNA preparations used for sequencing. *Nucleic Acids Res*. **21**, 1677–1678 (1993).
3. Bachmann, B. J. Pedigrees of some mutant strains of *Escherichia coli* K-12. *Bacteriol. Rev*. **36**, 525–557 (1972).
4. Jensen, K.F. The *Escherichia coli* K-12 "wild types" W3110 and MG1655 have an rph frameshift mutation that leads to pyrimidine starvation due to low *pyrE* expression levels. *J. Bacteriol*. **175**, 3401–3407 (1993).
5. Casadaban, M. J. & Cohen, S. N. Analysis of gene control signals by DNA fusion and cloning in *Escherichia coli*. *J. Mol. Biol*. **138**, 179–207 (1980).
6. Patterson, T. A. & Dean, M. Preparation of high titer lambda phage lysates. *Nucleic Acids Res*. **15**, 6298 (1987).
7. Griffin, P. M. *et al.* Illnesses associated with *Escherichia coli* O157:H7 infections. A broad clinical spectrum. *Ann Intern Med.* **109**, 705–712 (1988).
8. [Loś, J. M](https://www.ncbi.nlm.nih.gov/pubmed/?term=Lo%C5%9B%20JM%5BAuthor%5D&cauthor=true&cauthor_uid=20070366)., [Loś, M](https://www.ncbi.nlm.nih.gov/pubmed/?term=Lo%C5%9B%20M%5BAuthor%5D&cauthor=true&cauthor_uid=20070366)., [Wegrzyn, A](https://www.ncbi.nlm.nih.gov/pubmed/?term=Wegrzyn%20A%5BAuthor%5D&cauthor=true&cauthor_uid=20070366)., [Wegrzyn, G](https://www.ncbi.nlm.nih.gov/pubmed/?term=Wegrzyn%20G%5BAuthor%5D&cauthor=true&cauthor_uid=20070366). Hydrogen peroxide-mediated induction of the Shiga toxin-converting lambdoid prophage ST2-8624 in *Escherichia coli* O157:H7. *FEMS immunology and medical microbiology* **58 (3)**, 322–329; 10.1111/j.1574-695X.2009.00644.x (2010).
9. Beutin, L., Montenegro, M. A. & Orskov, I. Close association of verotoxin (Shiga-like toxin) production with enterohemolysin production in strains of *Escherichia coli*. *J. Clin. Microbiol*. **27**, 2559–2564 (1989).
10. Perna, N. T. *et. al*. Genome sequence of enterohaemorrhagic *Escherichia coli* O157:H7. *Nature* **409**, 529–533 (2001).
11. Januszkiewicz, A., Rastawicki, W. Molecular characterization of Shiga toxin-producing *Escherichia coli* strains isolated in Poland. [*Pol J Microbiol*.](https://www.ncbi.nlm.nih.gov/pubmed/29334059) **65**, 261–269 (2016).
